# Comparing Visual Search Efficiency Across Different Facial Characteristics

**DOI:** 10.3390/vision9040088

**Published:** 2025-10-15

**Authors:** Navdeep Kaur, Isabella Hooge, Andrea Albonico

**Affiliations:** Department of Psychology, University of the Fraser Valley, 33844 King Rd, Abbotsford, BC V2S 7M7, Canada; navdeep.kaur22@student.ufv.ca (N.K.); isabella.hooge@student.ufv.ca (I.H.)

**Keywords:** visual search, face processing, emotion, response times

## Abstract

Face recognition is an important skill that helps people make social judgments by identifying both who a person is and other characteristics such as their expression, age, and ethnicity. Previous models of face processing, such as those proposed by Bruce and Young and by Haxby and colleagues, suggest that identity and other facial features are processed through partly independent systems. This study aimed to compare the efficiency with which different facial characteristics are processed in a visual search task. Participants viewed arrays of two, four, or six faces and judged whether one face differed from the others. Four tasks were created, focusing separately on identity, expression, ethnicity, and gender. We found that search times were significantly longer when looking for identity and shorter when looking for ethnicity. Significant correlations were found among almost all tests in all outcome variables. Comparison of target-present and target-absent trials suggested that performance in none of the tests seems to follow a serial-search-terminating model. These results suggest that different facial characteristics share early processing but differentiate into independent recognition mechanisms at a later stage.

## 1. Introduction

Face recognition is an essential skill that enables people to make quick social judgments and extract social information from others based solely on the appearance of their face. Although the identity of the face is often considered the most prominent information that can be extracted from a face, faces also carry lots of other information (such as expression, ethnicity, and age) that allow us to identify other important information for communication and survival, such as understanding and interpreting the mood and intentions of others. The importance of faces has also been suggested as the reason for their specificity as a visual stimulus [1]. As additional evidence of the specificity of face processing, the ability to recognize faces can be selectively disrupted, leading to prosopagnosia. This impairment can be acquired, after brain damage [2], or developmental in the absence of any brain injury [3]. Most individuals with prosopagnosia report that their face recognition deficits also lead to severe social difficulties, such as anxiety, fear, and avoidance of social situations [4].

Different models of face recognition have been proposed, trying to explain how faces are processed in the human brain. Among those, one of the most prominent models is the Bruce and Young model [5]. This is a stage-based model of face perception that suggests that the process of recognizing a face involves several independent, sequential stages, including creating a structural representation of the face (structural encoding), comparing the structural representation to stored faces (face recognition units—FRUs), accessing biographical information associated with the face (personal identity nodes—PINs), and generating the person’s name (name retrieval). Critically, the model also suggests that separate routes exist for processing identity, emotion, and other facial attributes, such as those related to speech and eye gaze, so that the processing of a face’s identity and other facial characteristics is independent of each other [5].

A second model of face recognition was proposed by Haxby, Hoffman, and Gobbini [6]. This is a neuroanatomical model that describes face perception as supported by a distributed neural system with a core system and an extended system. The core system consists of three main regions: the inferior occipital gyrus (involved in the early perception of facial features), the lateral fusiform gyrus or fusiform face area (FFA) (processing invariant aspects of faces, such as identity), and the superior temporal sulcus (STS) (processing changeable aspects of faces, such as gaze, expression, and lip movement). The extended system includes additional regions that interact with the core, depending on the type of social or cognitive information required, such as the amygdala for emotional response and the anterior temporal cortex for personal identity and biographical knowledge. Critically, this model also suggests that the processing of the facial identity and other changeable characteristics of a face is (at least partially) independent [6].

The dissociation between face identity and expression or other changeable characteristics of a face is also supported by neuroimaging [7] and behavioral studies [8,9], even though some authors have claimed that the clear evidence for the separation between the processing of facial identity and expression might also be explained by different frameworks, other than the independence of the corresponding visual pathways [10]. Deficits in the processing of identity and other changeable aspects of the face seem to also be dissociated in acquired prosopagnosia [11,12]. Whether this is also true about developmental prosopagnosia is less clear, with mixed results [13,14,15]. One of the possible explanations for these mixed results is that most prior reports did not evaluate identity and expression processing in the same manner.

So, the present study aimed to compare the processing of different facial characteristics by using the same paradigm. To do so, we used a visual search task, in which participants looked for targets among distractors. Such tasks allow us to examine the speed and accuracy of facial recognition depending on the number of stimuli on the screen [16]. Changes in accuracy and response times as a function of the set size are measures of the search performance and have been used to investigate many aspects of perceptual processing [17] and the relationship between vision and attention [18]. Initially, it was proposed that visual search used either a serial or parallel strategy [17], but subsequent work suggests that this is probably more of a continuum than a dichotomy [16,19]. In this report, we showed participants an array of several stimuli (i.e., two, four, or six) and asked them to indicate if one of the stimuli differed from the others. We created four different tests: one in which participants were asked to make a judgment about the identity of the faces, one in which they were asked to judge the expression of the faces, one in which they had to judge the ethnicity of the faces, and a last one in which they had to judge the gender of the faces. Our goal was to investigate whether searching for different facial characteristics leads to different performance and search efficiencies.

## 2. Method

### 2.1. Participants

Ninety-nine participants were recruited (mean age = 35.3; s.d. = 10.9; range, 19–69; 49 males and 50 females) and tested. Eighty-seven of them (mean age = 35.1; s.d. = 11.0; range, 19–69; 43 males and 44 females; eight left-handed and one ambidextrous) met the performance criteria (see below for a detailed description of the exclusion criteria), confirming that they understood and executed the task correctly. All participants were native English speakers and had normal or corrected-to-normal vision. Most of the participants identified themselves as white (*n* = 29) or black (*n* = 26), with the remaining participants identifying as South Asian (*n* = 18), Pacific Islander (*n* = 9), Native American (*n* = 1), or other (*n* = 4).

To determine sample size, a power analysis was conducted using G*Power 3.1 [20] for a repeated-measure ANOVA (within factors) comparing the different tasks; a sample size of $\eta_{p}^{2}$ = 0.675, reported in [21], for set size effects was used in the power analysis: this implied that a sample size of 12 participants would be required to detect a difference in performance between the tasks with 95% probability. Because the effect size found in the present study was smaller ($\eta_{p}^{2}$ = 0.262), we also conducted a second power analysis with this new value, which implied that a sample size of 51 participants would be required to detect the effect with 95% probability. Thus, our sample size was well above the sample size estimate.

All participants were reimbursed CAD 5 for their time. The protocol was approved by the Institutional Review Boards of the University of the Fraser Valley, and all subjects gave informed consent in accordance with the principles of the Declaration of Helsinki.

### 2.2. Stimuli

**Figure 1:**
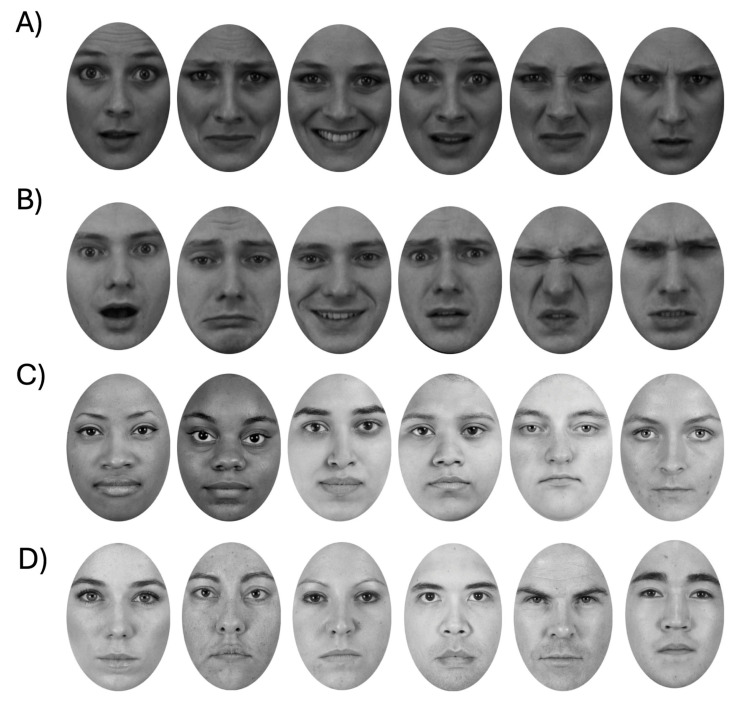
Examples of stimuli used in the tests: (**A**) six different pictures of the same face identity used in the identity test (from left to right: surprise, sadness, happiness, fear, disgust, and anger); (**B**) six different pictures of the same face identity used in the expression test (from left to right: surprise, sadness, happiness, fear, disgust, and anger); (**C**) six different identities used in the ethnicity tests (first two represent black, middle two Indian, and last two white ethnicity); (**D**) six identities used in the gender test.

Four sets of stimuli were used, once each for the identity, emotion, gender, and ethnicity conditions. To minimize contributions from low-level image matching, we had images vary not only in the primary dimension relevant to the test, but also in other irrelevant dimensions (Figure 1). Thus, while the identity test required subjects to report on facial identity, images also varied in facial expression. For the expression, gender, and ethnicity tests, subjects reported on the expression, gender, and ethnicity of the face, ignoring the identity of the face. All stimuli were taken from the Karolinska Directed Emotional Faces (KDEF) database [22] and the Chicago Face Database (CFD) [23]. The KDEF database was chosen because it includes multiple pictures of the same individual depicting different emotional expressions, while the CFD was chosen because it includes pictures of individuals from different ethnic backgrounds.

For the identity test, twelve identities (six females and six males) were selected. For each identity, we obtained six images, each depicting a different expression: anger, disgust, fear, happiness, sadness, and surprise. Similarly, for the expression test, we selected twelve different identities (six females and six males), each depicted with six different expressions: anger, disgust, fear, happiness, sadness, and surprise. For the ethnicity test, twelve identities (six females and six males) were selected for each ethnicity, namely, white, black, and Indian (as identified in the Chicago Face Database). For the gender test, twelve identities (six females and six males) were used. The identities used in each test were different to avoid excessive familiarization with the stimuli. Each face image was then converted to grayscale and cropped using Adobe Photoshop CC 2014 so that each image was placed within an oval aperture of 525 × 750 pixels, thereby occluding external cues such as hair and ears. To ensure that stimuli fit on the screen, they were presented at 35% of their original size.

### 2.3. Procedure

The tests were controlled by TESTABLE (https://www.testable.org, subscription date is 30 August 2023). Participants were instructed to always conduct the experiment alone and in a quiet environment with the screen at an arm’s length distance. The screen resolution was calibrated using a credit card as a standardized unit of measurement before the experiment began, to ensure consistency in stimulus size.

There were four different tests: one in which participants were required to search for facial identity, one for facial expression, one for ethnicity, and one for gender. The test order was counterbalanced across participants.

**Figure 2:**
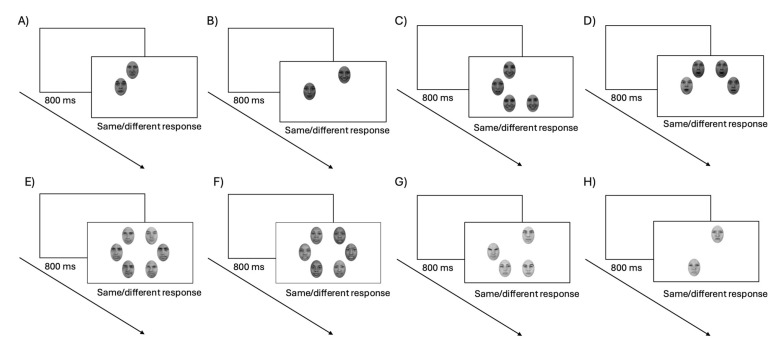
Examples of both *same* and *different* trials for the identity, expression, ethnicity, and gender tests. (**A**,**B**) depict a *different* and a *same* trial for the identity test. (**C**,**D**) depict a *different* and a *same* trial for the expression test. (**E**,**F**) depict a *different* and a *same* trial for the ethnicity test. (**G**,**H**) depict a *different* and a *same* trial for the gender test.

Participants performed a same–different task, and the procedures of each test and its trials were similar for the four tests. All stimuli were presented against a white background, and responses were collected using a keyboard. Each trial (Figure 2) started with a blank screen, followed 800 ms later by the stimulus display. The target screen displayed an array of faces, with a possible set size of two, four, or six stimuli. In the identity test, the images were all from the same person for the same trials, whereas for the different trials, one image depicted a different identity. In both the same and different trials, the expression varied between all images (so that the target face in the different trials always had a different expression than the other stimuli, and in the same trials, no expression was repeated within the same trial). In the expression test, the same trials all had the same expression, whereas the different trials had one face with a different expression, with each stimulus depicting a different identity. In the ethnicity test, the same trials had all faces of the same ethnicity (white, black, or Indian), while different trials had one face with a different ethnicity than the others, with each stimulus depicting a different identity. In the expression and ethnicity tests, all possible combinations of expression and emotions were tested in the same number. Finally, in the gender task, the same trials had all faces of the same gender (female or male), while the different trials had one face depicting a different gender than the others, again with each stimulus depicting a different identity. Participants were asked to press the ‘S’ key on the keyboard if all the stimuli were of the same, and the ‘D’ key if one image differed from the rest. The target screen remained on the screen until the participant’s response.

Each test began with a practice phase of six trials that familiarized the participants with the test (two trials for each set size were shown in the practice phase, one each for the same and different conditions). During the practice but not the experimental trials, participants received feedback after each response. Participants needed to achieve an accuracy of at least 80% in the practice phase to proceed to the actual test; if this criterion was not met, practice was repeated.

A total of 54 trials were completed for each test: 36 different trials in which one of the stimuli differed from the others in the corresponding dimension (i.e., identity, expression, ethnicity, or gender), and 18 same trials in which one of the stimuli was different from the others in the relevant dimension. Participants were not informed of this proportion. Trials were equally distributed among the different possible set sizes (i.e., 2, 4, or 6 stimuli). The presentation of the stimuli was balanced so that each stimulus appeared an equal number of times in each test. The target on different trials was located as evenly as possible across all stimulus positions of the set. We recorded both accuracy and RT, defined as the time between the appearance of the stimuli and the participant’s keypress. The entire study took around 15–25 min for participants to complete.

### 2.4. Data Analysis

Chance performance in our task is 50% (only two possible answers: same or different), and with 54 trials per task, the upper 95% limit of our 50% chance performance is 63%. (this performance threshold was calculated by using the following formula: *p* $\pm \;Z_{1-\alpha /2}*\;\surd (\frac{p\left(1-p\right)}{n})$, where *p* is the chance level, and *n* is the number of trials in the test). We used this 63% cut-off on overall performance (all conditions combined) as a measure of participants’ ability to understand and execute the task so that participants who were below this threshold were excluded from the analysis (and examination of the individual data from those participants confirmed that they did not properly engage in the task, as visible from the large number of missing responses or multiple buttons pressed). This excluded eleven of the ninety-nine original participants, resulting in a final sample of eighty participants (see above).

For each of the four tests, we analyzed four outcome variables, two reflecting accuracy and two being temporal measures. For accuracy, we calculated the mean accuracy for both the same and different trials together. Next, we used the hits (participants responded ‘different’ on target-present trials) and false alarms (participants responded ‘different’ on target-absent trials) to calculate d’, a criterion-free index of discrimination sensitivity that reflects the participants’ ability to detect a particular signal [24]. For temporal measures, we used only correct trials and first calculated the mean response time for each block in each subject. Next, we calculated the set size effect, which was the slope of the linear regression between the response time and the number of objects in the array, indexing performance as a function of perceptual processing load.

First, for each of the four variables, we submitted the data to a one-way repeated-measures ANOVA, with tests (identity, expression, ethnicity, and gender) as the within-subject factors. This was done to explore whether participants’ performance varied across the different tasks. Significant differences were further explored by Bonferroni post hoc multiple comparisons (corrected *p*-values are reported), in particular comparing upright and inverted performance for each stimulus type. Effect sizes were measured by computing the Partial Eta Squared ($\eta_{p}^{2}$). Second, to investigate whether common mechanisms were employed for the different facial characteristics, we performed correlational analyses to determine whether the results of the different tests were correlated with each other. Third, to investigate the termination rule adopted by the participants in the different tasks, we compared the results of the same and different trials. For each participant, we computed the natural logarithm of the ratio of the set size effect for the same over the different trials. A standard serial self-terminating search model predicts a ratio of 2 when exhaustive search of all stimuli is needed to reach a correct decision on same trials, whereas search on different trials can be terminated by discovery of the target, which will occur on average about halfway through searching [19,25]. Therefore, we assessed the different tests to determine whether this value was significantly different from ln(2).

## 3. Results

### 3.1. Accuracy and d’

**Figure 3:**
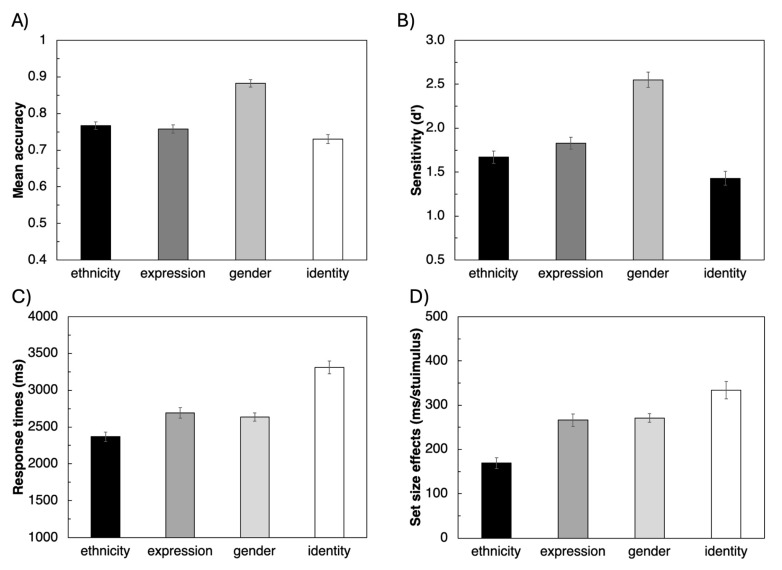
Accuracy and temporal outcome variables for the four tests. (**A**) Mean accuracy data, (**B**) sensitivity (d′) data, (**C**) mean response time data, and (**D**) set size effects. Error bars indicate one standard error. Significant pairwise differences are reported in the text.

The ANOVA on the accuracy data revealed a significant effect of the test (F(3, 258) = 51.0, *p* < 0.001, $\eta_{p}^{2}$ = 0.372; Figure 3), and post hoc comparison revealed that accuracy was significantly higher for the gender test (M = 0.88, SE = 0.01) than for the ethnicity (M = 0.77, SE = 0.01; *p* < 0.001), expression (M = 0.76, SE = 0.01; *p* < 0.001), and identity tests (M = 0.73, SE = 0.01; *p* < 0.001). Accuracy in the ethnicity tests was also significantly higher than in the identity test (*p* = 0.022).

The ANOVA on the d-prime revealed very similar results. The main effect of the test was significant (F(3, 258) = 52.6, *p* < 0.001, $\eta_{p}^{2}$). = 0.379; Figure 3). Performance was significantly better for the gender test (M = 0.2.55, SE = 0.09) than for the ethnicity (M = 1.67, SE = 0.07; *p* < 0.001), expression (M = 1.83, SE = 0.0.07; *p* < 0.001), and identity tests (M = 1.43, SE = 0.0.08; *p* < 0.001). Performance in the ethnicity and expression tests was also significantly better than in the identity test (respectively, *p* = 0.031 and *p* < 0.001).

### 3.2. Response Times and Set Size Effects

The ANOVA on the response times revealed a significant effect of the test (F(2.68, 230.24) = 74.3, *p* < 0.001, $\eta_{p}^{2}$ = 0.464) because of significantly faster response times for the ethnicity test (M = 2369 ms, SE = 64 ms) compared to all other conditions (all ps < 0.001) (since Mauchly’s test revealed a violation of the sphericity assumption (Mauchly’s W = 0.847, *p* = 0.015), the degrees of freedom reported are corrected according to the Greenhouse–Geisser method) (Figure 3). Response times for the gender (M = 2636 ms, SE = 56 ms) and expression (M = 2692 ms, SE = 74 ms) tests were also faster than those for the identity test (M = 3311 ms, SE = 87 ms; both ps < 0.001). Only the response times in the gender and expression tests did not significantly differ from each other (*p* = 1.000).

**Figure 4:**
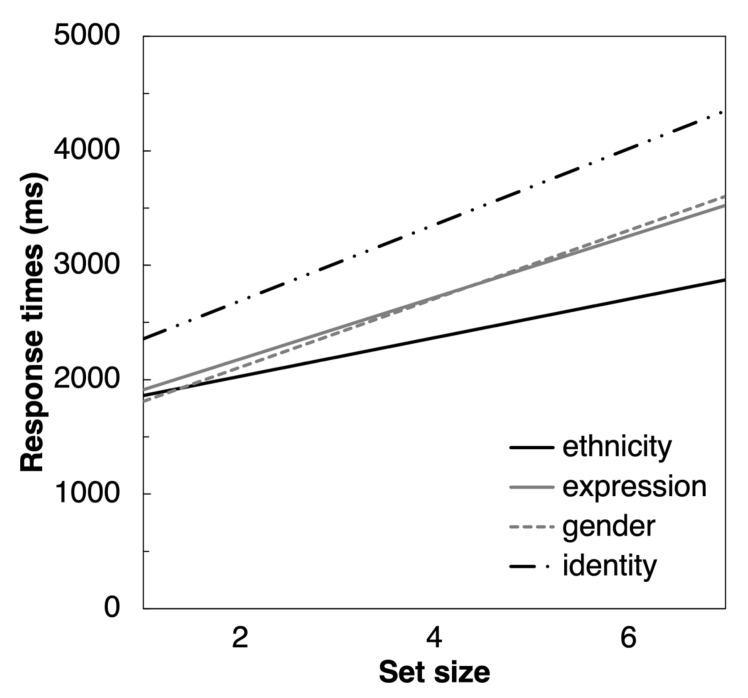
Mean response times as a function of the set size for all four tests.

Similar results were found in the ANOVA on the effect size. The main effect of the test was significant (F(2.37, 203.63) = 30.6, *p* < 0.001, $\eta_{p}^{2}$ = 0.262; Figure 3) (since Mauchly’s test revealed a violation of the sphericity assumption (Mauchly’s W = 0.693, *p* = < 0.001), the degrees of freedom reported are corrected according to the Greenhouse–Geisser method). Set size effects (Figure 4) were significantly smaller for the ethnicity test (M = 169 ms/stimulus, SE = 12 ms/stimulus) compared to all other conditions (all *p* < 0.001). Set size effects for the expression (M = 266 ms/stimulus, SE = 14 ms/stimulus) and gender tests (M = 271 ms/stimulus, SE = 10 ms/stimulus) were also smaller than those for the identity tests (M = 334 ms/stimulus, SE = 20 ms/stimulus; respectively *p* = 0.018 and *p* = 0.010). Only the set size effects in the gender and expression tests did not significantly differ from each other (*p* = 1.000).

### 3.3. Correlations

Correlation between different tasks may suggest common mechanisms that operate on different stimuli (Table 1). We found consistent and significant correlations among most tests in all four outcome variables. The only exceptions are the identity and expression tests, whose correlations seem to be less reliable.

### 3.4. Target-Absent (Same) Versus Target-Present (Different) Trials

Our analyses above report results in which mean accuracy, d’; mean response time; and object set size effect are computed by combining the data from both target-present and target-absent trials. Hence, errors in our tasks include both misses and false alarms, consistent with low-threshold theories of search [26]. In contrast, false alarms do not occur in high-threshold theory. Thus, set size effects such as those we found do not occur in high-threshold theory but are characteristic of low-threshold theories [27].

Table 2 shows that the set size effects are larger for the same trials compared to different trials in all tests. Our set size effects are much larger than the typical 20–60 ms/item reported for searches with simpler visual stimuli and most likely incorporate the time to execute searching saccades [19]. A ratio of 2 is predicted by termination rules in the standard serial self-terminating search model, in which exhaustive search is needed to reach a correct decision on target-absent trials, while a search on target-present trials can be terminated by discovery of the target, which will occur on average about halfway through scanning [19,25]. Participants showed values around 1.5 or lower for all tests. Lower ratios of around 1.5 have been seen in searches with premature termination on target-absent trials, possibly due to a contribution of guidance from parallel pre-attentive mechanisms [25]: inspection of the data in Table 2 seems to confirm this hypothesis for the ethnicity test, where set size effects are similar for the same and different trials. For the other tests, the reduced ratio seems to be due to increased response times for different trials, particularly in the identity test (the only ratio that did not differ from the ln(1.5)).

## 4. Discussion

Our results showed that the gender test was associated with higher accuracy and sensitivity, while the identity test had the lowest accuracy and sensitivity, suggesting that finding the gender target might be easier than looking for other facial characteristics, with identity being the most challenging condition. A significant effect of the test was also found on the temporal variables. Although the analysis of mean RT and set size effects produced similar results, among the two temporal variables, set size effects may more accurately reflect the perceptual processing load specific to faces [28]. Indeed, mean RT inevitably includes factors such as general processing speed and motor latency that are not specific to face processing. We found that set size effects were longer when searching for identity and smaller when searching for a different ethnicity, with expression and gender in between.

These findings suggest that the search for identity is less efficient than the searches for other facial characteristics, possibly reflecting a dissociation in the processing of these different facial characteristics. A few studies have examined visual search using faces as stimuli. Set size effects for facial identity or expression are typically larger than those for simpler items, indicating the use of a serial-search model. In particular, previous studies have found similar search slopes for identity and expression [21,29], whereas we found longer set size effects for identity than for expression, as well as for gender and ethnicity. Our different results seem to be consistent with the independence of the invariant and changeable aspects of a face, as postulated by some of the main models of face recognition [5,6]. Indeed, the invariant and changeable aspects of a face seem to have distinct representations in the temporal lobe [30]. Some studies have also suggested that processing invariant and changeable aspects of faces relies on different cues, with invariant aspects, such as identity, being more based on surface-based cues, whereas changeable aspects, such as expression, rely more on edge-based cues [31]. Relying on different facial cues might explain the different search times we found in our tests. This hypothesis seems to be particularly relevant for the smaller set size effects we found in the ethnicity test. Previous studies have demonstrated that ethnicity can guide attention in visual search. For instance, Otten [32] showed that black target faces were detected more efficiently than white target faces, but that this advantage can be affected by the facial expression depicted in the target face. Another study [33] found faster search times for other-race-than-own-race faces in Chinese participants but no search asymmetry in Caucasian participants, suggesting that the ethnicity of a face might not be a visual basic feature; this finding suggests that the salient color and brightness features of faces could be the source of the smaller search slopes when looking for a particular ethnicity [33]. Familiarity might play a role, as also suggested by a study that found a smaller set size effect when participants are searching for their own face [34]. One limitation of our study is that we did not take into consideration the participants’ ethnicities, which have also been shown to modulate visual search for faces of different ethnicities [33]; future studies should also investigate the interaction between the stimuli and participants’ ethnicities in visual search tasks.

The set size effects for gender and expression were increased compared to the ethnicity one, even if still significantly reduced compared to the set size effect for identity. A possible explanation for this partial increase in the gender and expression tests is that in our expression and gender tests, we averaged data from the six basic expressions and the two genders. Although there is evidence that these six basic expressions are recognized and produced similarly regardless of the cultures [35], some studies have suggested that not all emotions are processed similarly. For instance, neuroimaging [36] shows that each emotion we may experience, such as fear or happiness, has distinct patterns of brain activity. Kragel and colleagues (2016) [36] also found that even when individuals are not thinking of a specific emotion, for example, when resting or not really thinking at all, their brains still show brain activity that reflects emotional states. Recent work has demonstrated that there is also a difference between individuals in the rate at which each person can process emotions [37]. They found that different functional connectivity can predict how fast someone can identify emotional expressions. On a behavioral level, others [38] have found that positive facial expressions are recognized significantly faster than negative ones. Regarding visual search tasks, several studies have found that different emotions lead to different search slopes: for instance, smaller set size effects have been found when searching for a negative facial expression than for a positive one [39], and when looking for a threatening emotion compared to a neutral or non-threatening one [40]. Similarly, it has been shown that the efficiency of face gender categorization changes depending on the gender of the face, suggesting that recognition of male and female faces might be different cognitive processes [41]. One limitation, and possible confounding factor, of the present study is that the stimuli used in the different tests slightly varied in resolution and average luminance since they are from different databases. Matching the average luminance across all stimuli was not possible, as this would have distorted some of the faces (particularly those in the ethnicity condition), thereby affecting the task’s difficulty. However, it is unlikely that our results are due to the luminance difference across the stimuli. The stimuli for the identity and expression tasks were chosen from the KDEF database, while the ones for the ethnicity and gender tasks were from the CFD. Our results, though, did not cluster in this way: indeed, the accuracy and sensitivity results showed that performance in the expression test was similar to performance in the ethnicity tests, despite the different stimuli used. Similarly, analysis of the temporal variables (i.e., response times and set size effects) showed that performance in the expression task was similar to that in the gender task, again, despite the different stimuli used.

Our correlational analyses revealed significant correlations among almost all tests in all outcome variables. Such correlations might suggest that, despite the different search slopes in the four tests, there is some shared processing among identity, gender, ethnicity, and expression. However, shared processes could be either general perceptual abilities or face-specific mechanisms. Some degree of correlation is expected given that we used the same paradigm for all four tests, so some general abilities might be partially responsible for the correlations that we found. Set size effects, though, are more likely to isolate a temporal effect more specific to face processing from more general perceptual effects, compared to response times or accuracy and sensitivity. For this variable, we still found significant correlations, thus suggesting that, even when general perceptual abilities are taken into account, there is still some shared processing among the four face characteristics we considered. This result does not exclude the possibility that the processing of identity, emotion, gender, and ethnicity is a separate process: indeed, it might reflect the fact that this separation occurs only after a first shared stage of visual processing. In the Bruce and Young model [5], for instance, inputs for the expression analysis are provided by view-centered descriptions, which are also relevant for the structural encoding of a face. Similarly, in Haxby and colleagues’ model [6], the early perception of facial features in the inferior occipital gyri is relevant for the processing of both the invariant and changeable aspects of the face.

Finally, we compared the participants’ performance in the same and different trials. Indeed, the ratio of set size effects between the two is informative of the termination rules adopted. A standard serial self-terminating search model predicts a ratio of 2 [19,25]. In the present study, we found that none of the ratios [21,28,42] were close to the predicted value of 2, unlike what we found in previous studies. Most of the ratios in the present study were close to a value of 1.5, which has been previously reported in searches with premature termination on target-absent (same) trials, possibly due to a contribution of guidance from parallel pre-attentive mechanisms [25]. Inspection of our data seems to confirm this hypothesis for the ethnicity test, but not for the expression and gender ones, where the decreased ratio could be due to longer search times in the different trials. Finally, the ratio for the identity test was closer to a value of 1. A set size ratio of 1 violates the prediction for self-terminating serial search, but why this should be is not clear. One suggested possibility is a more efficient search on the same trials. However, our data does not seem to go in this direction. Indeed, it seems more likely that the reduced ratio in the identity test is due to increased search times in the different trials, possibly suggesting that the discovery of the target may not terminate the search for facial identity. One possible way to disentangle these different possibilities would be for future studies to record eye movements: indeed, previous studies have investigated optimal eye movement patterns in visual search tasks [43,44] and have shown that eye movement can provide important information on how faces with different characteristics impact attentional orienting and processing [45].

In conclusion, our results indicate increased set size effects in visual search for identity compared to expression, ethnicity, and gender, suggesting at least a partial independence in the processing of such facial characteristics. Our correlational analysis, though, suggests that such separation might arise only after a shared initial stage of visual processing. Comparison between the same and different trials revealed that a self-terminating serial search might not apply to visual search tasks involving faces, regardless of the facial characteristic that has to be detected.

## Figures and Tables

**Table 1:** Correlations between the ethnicity, expression, gender, and identity search results.

**Accuracy**					**d Prime**				
	ethnicity	expression	gender	identity		ethnicity	expression	gender	identity
ethnicity					ethnicity				
expression	**0.321** ** **(0.119, 0.498)**				expression	**0.297** ** **(0.092, 0.478)**			
gender	**0.290** ** **(0.084, 0.472)**	**0.220** * **(0.009, 0.411)**			gender	**0.289** ** **(0.083, 0.471)**	0.209 (−0.002, 0.402)		
identity	**0.409** *** **(0.217, 0.571)**	0.204 (−0.007, 0.397)	0.200 (−0.011, 0.394)		identity	**0.398** *** **(0.205, 0.562)**	0.129 (−0.084, 0.331)	**0.214** * **(0.004, 0.407)**	
**Response time**					**Set size effect**				
	ethnicity	expression	gender	identity		ethnicity	expression	gender	identity
ethnicity					ethnicity				
expression	**0.667** *** **(0.531, 0.770)**				expression	**0.403** *** **(0.210, 0.565)**			
gender	**0.579** *** **(0.420, 0.704)**	**0.627** *** **(0.480, 0.740)**			gender	**0.304** ** **(0.100, 0.484)**	**0.283** ** **(0.076, 0.466)**		
identity	**0.563** *** **(0.400, 0.692)**	**0.587** *** **(0.429, 0.710)**	**0.577** *** **(0.417, 0.703)**		identity	**0.383** *** **(0.188, 0.549)**	0.181 (−0.031, 0.377)	**0.277** ** (0.070, 0.460)	

Values in parentheses indicate the 95% confidence interval for the correlation index. Bold indicates significant values. * indicates *p* < 0.05. ** indicates *p* < 0.01. *** indicates *p* < 0.0001.

**Table 2:** Results for ‘same’ (target absent) and ‘different’ (target present) trials. Bold indicates significant values.

	Ethnicity		Expression		Gender		Identity	
Controls	Mean	sd	Mean	sd	Mean	sd	Mean	sd
same trials	199	178	333	219	363	156	400	318
different trials	147	145	230	147	221	106	315	229
ratio	1.35		1.45		1.64		1.27	
z(1)	−7.89		−6.53		−3.98		−9.19	
p(1)	**<0.001**		**<0.001**		**<0.001**		**<0.001**	
z(2)	−1.95		−0.67		1.72		−3.16	
p(2)	0.051		0.501		0.085		**0.001**	

z(1) and p(1) are the z-score and the probability that the natural logarithm of the average ratio differs from a value of ln(2); z(2) and p(2) are the z-score and the probability that the natural logarithm of the average ratio differs from a value of ln(1.5).

## Data Availability

The data presented in this study are available on request from the corresponding author due to ethical restrictions.
